# Cathepsin C Restricts Influenza A Virus Replication and Is Associated with Suppression of the PI3K-AKT Signaling Pathway

**DOI:** 10.3390/v18060641

**Published:** 2026-06-03

**Authors:** Yansheng Zhu, Lanlan Si, Zhongzhong Cao, Huiyang Song, Aiqing Yang, Xia Wang, Yifei Qiu, Chengming Gao, Gangqiao Zhou, Pengbo Cao

**Affiliations:** 1 College of Life Sciences, Anhui Medical University, Hefei 230032, China; yszhu810@163.com; 2 Graduate School, Guangzhou Medical University, Guangzhou 511436, China; sll1900@163.com; 3 College of Life Sciences, Hebei University, Baoding 071002, China; 19515660618@163.com; 4 Academy of Military Medical Sciences, Beijing 100850, China; song_hickory@163.com (H.S.); yan_gaiqing_ok@126.com (A.Y.); 18589558796@163.com (X.W.); 3180101060@zju.edu.cn (Y.Q.); gchengming1988@163.com (C.G.); 5 State Key Laboratory of Medical Proteomics, National Center for Protein Sciences at Beijing, Beijing 100850, China

**Keywords:** H1N1, Cathepsin C, PI3K-AKT pathway, apoptosis, inflammation

## Abstract

Cathepsin C (CTSC) is a major lysosomal cysteine protease characterized by its involvement in multiple essential pathological processes associated with various viral infections and pathogenesis, such as influenza virus and coronavirus. However, the antiviral spectrum of CTSC and the molecular mechanisms underlying its activity remain to be fully elucidated. In this study, we demonstrate that CTSC significantly inhibits the infection of influenza A virus (IAV) H1N1 both in vitro and in vivo. Mechanistically, the antiviral function of CTSC is associated with attenuation of the PI3K-AKT signaling pathway, thereby inducing cell apoptosis and reducing inflammation, which ultimately limits the virus’s ability to hijack host resources. Taken together, our findings highlight the crucial role of CTSC in defense against H1N1 by targeting PI3K-AKT pathway and suggest a prospective antiviral target against the infection of H1N1.

## 1. Introduction

Influenza A virus (IAV) remains a persistent global health threat, responsible for substantial annual morbidity and mortality. Its rapid evolution and zoonotic potential consistently heighten the risk of pandemics [1]. Although current antiviral therapies primarily target viral components, the emergence of resistant strains highlights an urgent need for strategies that focus on host factors, a direction less likely to drive resistance [2]. Host factors play essential roles across multiple stages of the viral life cycle [3], and their modulation represents a promising approach for developing broad-spectrum antiviral agents [4].

Cathepsin C (CTSC), a key member of the lysosomal cysteine protease family, exerts its fundamental biochemical function as a chloride-dependent dipeptidyl peptidase I (DPPI) by specifically catalyzing the removal of dipeptides from the N-terminus of substrate proteins, thereby participating in the activation of granzymes and serine proteases in immune cells [5]. Recently, its regulatory functions in viral infection, such as Severe Acute Respiratory Syndrome Coronavirus 2 (SARS-CoV-2) and Influenza A virus subtype H1N1, have gained increasing recognition [6,7]. Li et al. performed the first GWAS on the severity of COVID-19 in the Chinese population and identified the chromosome 11q14.2 locus associated with CTSC as playing a crucial role in the genetic susceptibility to COVID-19 severity [8]. However, the specific role and underlying mechanisms of CTSC in viral infection, particularly in host defense against IAV, remain poorly understood.

Host factors are critical determinants of IAV infection, governing viral replication through several major mechanisms, including viral entry (e.g., receptor recognition), innate immune responses (e.g., interferon signaling), intracellular trafficking, and the regulation of apoptosis. Among these, the delicate balance between host cell survival and programmed cell death is particularly important for a productive infection [9].

Recent studies have illuminated how IAV intricately manipulates this balance. A key strategy is the early activation of pro-survival signaling pathways to inhibit premature apoptosis. The PI3K-AKT axis, a classic pro-survival and anti-apoptotic cascade, is activated early during infection by various viruses, including IAV, to suppress host cell apoptosis. This ensures the preservation of the cellular machinery long enough for efficient viral replication [10]. Consistently, accumulating evidence demonstrates that the modulation of the PI3K-AKT pathway profoundly impacts IAV replication [11].

In the context of apoptosis-related research, further studies have revealed that IAV also finely regulates cell death by modulating mitochondrial pathways and the expression of Bcl-2 family proteins. The PI3K/AKT signaling pathway is established as a key player in IAV infection, where its activation inhibits premature apoptosis to support viral replication [12]. Furthermore, host cellular kinases, including PI3K, are therefore central to regulating IAV infection, with roles in modulating viral entry and replication [13]. In this study, we demonstrate that CTSC establishes a potent host-intrinsic defense barrier against H1N1 influenza both in vitro and in vivo. Our results indicate that CTSC suppresses PI3K-AKT–mediated survival signaling and promotes apoptosis in H1N1-infected human embryonic kidney (HEK293T) and respiratory epithelial (A549) cells. By clarifying a previously undefined antiviral role of CTSC in H1N1 infection, this study establishes a conceptual foundation for its potential as a host-directed therapeutic target.

## 2. Materials and Methods

### 2.1. RNA-Sequencing and Bioinformatics Analyses

Total RNA was extracted from A549 cells using TRIzol reagent (Invitrogen, 15596026, Carlsbad, CA, USA) according to the manufacturer’s instructions. RNA integrity was verified, and sequencing libraries were constructed and sequenced on an Illumina NovaSeq 6000 platform (PE150) (Illumina, San Diego, CA, USA). The raw reads were processed through a quality control pipeline to obtain clean reads. Clean reads were aligned to the human reference genome (GRCh38) using HISAT2 (v2.2.1) [14], and gene expression levels were quantified as counts by featureCounts (v2.0.6) [15]. Differential expression analysis between groups was performed using the DESeq2 package (v1.42.1) [16] in R, with genes meeting |log_2_(fold change)| > 0.585 and *p* value < 0.05 considered as significantly differentially expressed. Visualization of differential expression patterns was achieved through volcano plots (generated using the ggplot2 package (v3.5.2) [17]) and heatmaps (generated using the pheatmap package (Kolde R. pheatmap: Pretty Heatmaps. R package version 1.0.12. 2019. https://CRAN.R-project.org/package=pheatmap, accessed on 15 October 2025)). Functional enrichment was analyzed using the clusterProfiler package for Gene Ontology (GO) terms and Kyoto Encyclopedia of Genes and Genomes (KEGG) pathways [18]. Additionally, Gene Set Enrichment Analysis (GSEA) [19] was also conducted using the clusterProfiler package (v4.14.0) to identify significantly enriched biological pathways based on the Molecular Signatures Database (MSigDB v2026.1. Hs).

Publicly available RNA-seq datasets GSE156152 (BioProject: PRJNA656848, GPL20301, Illumina HiSeq 4000 (Homo sapiens) (Illumina, San Diego, CA, USA) and GSE255604 (GPL24676, Illumina NovaSeq 6000 (Homo sapiens) (Illumina, San Diego, CA, USA) [20] were downloaded from the Gene Expression Omnibus (GEO) database. GSE156152 contains RNA-seq data from 9 samples of 293T cells infected with wildtype (native) influenza A/WSN/1933 (H1N1) or with a mutant virus deficient in encoding fully functional NS1 (WSNdelNS1) to explore induced transcriptomic comparison between wildtype and mutant viral infections. GSE255604 contains RNA-seq data from 9 samples of A549 cells infected with H1N1 influenza virus and treated with mannose, examining how mannose remodels glucose metabolism to reduce inflammatory responses.

These datasets were re-analyzed following the same bioinformatics pipeline as described above. To integrate findings across datasets, a meta-analysis was performed using a random-effects model to identify consistently dysregulated genes and pathways, providing a more robust assessment of transcriptional changes across experimental contexts.

The RNA-sequencing data generated in this study will be deposited in the Gene Expression Omnibus (GEO) database upon acceptance.

### 2.2. Cell Lines

The human embryonic kidney cell line HEK293T, human alveolar basal epithelial carcinoma cell line A549 and the madin-darby canine kidney cell line MDCK were all obtained from the China Center for Type Culture Collection (CCTCC; Wuhan, China). All the cell lines were maintained in high glucose Dulbecco’s modified Eagle’s medium (DMEM; 11965118, Thermo Fisher Scientific, Waltham, MA, USA), supplemented with 10% fetal bovine serum (FBS; A5256701, Thermo Fisher Scientific, USA), 100 units/mL penicillin, and 100 units/mL streptomycin (15070063, Thermo Fisher Scientific, USA) under the conditions of 37 °C, 5% CO_2_, and a certain humidity.

### 2.3. Generation of the Ctsc-Knockout Mice

The heterozygous *Ctsc*-knockout (*Ctsc*-KO) mice were developed via CRISPR/Cas9-mediated deletion of exons 2–3 in the *Ctsc* gene using two single guide RNAs (sgRNAs) targeting adjacent intronic regions (sgRNA1: 5′-CAATAGAATATTGAGGAGGTGGG-3′, and sgRNA2: 5′-ACATAGAAGCCTTGCAGACTGGG-3′) (Listed in Supplementary Table S1). Following co-injection of in vitro-synthesized Cas9 mRNA with these sgRNAs into mouse zygotes, which were then implanted into pseudopregnant C57BL/6 female mice for embryo development. Genomic DNA extracted from 4-week-old *Ctsc*-KO mice tail biopsies using the Vazyme one Step Mouse Genotyping Kit (PD101-01; Nanjing, China) was subjected to PCR verification with genotype identification primers (Forward: 5′-GGAGCCCTTCTTCATCACC-3′, Reverse: 5′-TCTCCCTCTTGCAAAACTAATAAT-3′), confirming successful exon 2–3 deletion. All animal procedures were approved by the Animal Ethics Committee of Shanghai Model Organisms Center (Shanghai, China) and Academy of Military Medical Sciences (Beijing, China).

### 2.4. Viruses

Influenza A virus (IAV) strain A/Puerto Rico/8/1934 (H1N1; PR8) was propagated in MDCK cells and titrated by plaque assay. Viral stocks were prepared and confirmed by next-generation sequencing. All work with live H1N1 PR8 virus was conducted in BSL-2 facilities at the Beijing Academy of Military Medical Sciences, with viral stocks stored at the Guangzhou Customs District Technology Center BSL-3 Laboratory.

### 2.5. Antibodies

The following antibodies and reagents were used in this study: mouse anti-CTSC (sc-74590, Santa Cruz, Dallas, TX, USA); mouse anti-IAV NP (ab128193, Abcam, Waltham, MA, USA); mouse anti-Flag tag (MA1-142, Thermo Fisher Scientific, USA); rabbit anti- PI3 Kinase p85 (4257T, Cell Signaling Technology, Danvers, MA, USA); rabbit anti-phospho-PI3 Kinase p85 (17366T, Cell Signaling Technology, USA); rabbit anti-Akt (pan) (C67E7) (4691T, Cell Signaling Technology, USA); rabbit anti-phospho-Akt (Ser473) (D9E) (4060T, Cell Signaling Technology, USA); rabbit anti-mTOR (7C10) (2983T, Cell Signaling Technology, USA); rabbit anti-phospho-mTOR (Ser2448) (D9C2) (5536T, Cell Signaling Technology, USA); rabbit anti-β-Actin (AF5003, Beyotime, Shanghai, China).

### 2.6. Gene Knockdown and Overexpression Assays

For stable knockdown assays, the small hairpin RNA (shRNA) targeting human *CTSC* (*NM*_001114173.3) and a non-targeting control shRNA were synthesized by Tsingke Biotechnology Co., Ltd. (Beijing, China). The target sequences for human *CTSC* were as follows: sh*CTSC*-1: 5′-GCAGGAAAGTACGCCCAAGAT′ and sh*CTSC*-2: 5′-GATGACTTCCTCCACTACAAA-3′; the control shRNA sequence was 5′-GGCGCCGAACGGCGCACGGTT-3′ (Listed in Supplementary Table S1). For lentivirus production, HEK293T cells were co-transfected with the shRNA-expressing lentiviral plasmid and the packaging plasmids (psPAX2 and pMD2.G) using Lipofectamine™ 2000 (Invitrogen, USA). At 48 h post-transfection, the viral supernatant was harvested and used to infect target cells. Stable cell lines expressing the shRNA were established by 2 μg/mL puromycin selection for two weeks, and knockdown efficiency was confirmed by Western blotting.

To establish stable CTSC overexpression in HEK293T, A549, the cDNA encoding human *CTSC* (*NM*_001114173.3) was synthesized and cloned into a lentiviral expression vector (pLVX-CMV-3×Flag) by Tsingke Biotechnology Co., Ltd. (Beijing, China). The CTSC-containing construct was co-transfected with lentiviral packaging plasmids (psPAX2 and pMD2.G) into HEK293T cells using Lipofectamine 2000 (Invitrogen, USA), following the manufacturer’s instructions. Viral supernatants were collected 48 h post-transfection, filtered through a 0.45 μm membrane, and then applied to target cells at a 1:10 dilution in the presence of 8 μg/mL polybrene for 6 h. Transduced cells were selected with 2 μg/mL puromycin (ant-pr-1, InvivoGen, San Diego, CA, USA) for two weeks before assessing overexpression efficiency. All constructs were verified by Sanger sequencing and CTSC overexpression was confirmed by Western blot analyses.

### 2.7. Mice Experiments

Six to eight-week-old C57BL/6 mice, including wild-type (WT) and *Ctsc* knockout (*Ctsc*-KO) strains, weighing 18–22 g were used. Under light isoflurane anesthesia, mice were intranasally inoculated with 200 plaque-forming units (PFU) of H1N1 in 50 μL PBS. Mice were divided into two experimental groups: WT-infected and *Ctsc*-KO-infected. For survival analyses, five mice per group were monitored daily for body weight and survival until 14 days post-infection (dpi); moribund mice (defined by a weight loss exceeding 30% of initial body weight, severe lethargy, or dyspnea) were humanely euthanized, and the time of death was recorded. For tissue collection, an additional ten mice per group were used, with five mice from each group euthanized at 3 and 6 dpi, respectively. Lungs were aseptically harvested; the left lobe was fixed in 4% paraformaldehyde for subsequent paraffin embedding and hematoxylin and eosin (H&E) staining, while the right lobe was snap-frozen in liquid nitrogen and stored at −80 °C for viral titer determination.

All animal procedures were approved by the Institutional Animal Care and Use Committee of Academy of Military Medical Sciences (Beijing, China) and were conducted in accordance with relevant animal welfare guidelines. All H1N1-related experiments were performed in an Animal Biosafety Level 2 (ABSL-2) laboratory at the Academy of Military Medical Sciences.

### 2.8. Virus Infection

To assess the effect of CTSC on viral replication, control, CTSC-overexpressing, and CTSC-knockdown A549 and HEK293T cells were seeded in triplicate in 24-well plates. For H1N1 infection, cells were washed with PBS and incubated with virus at an MOI of 0.1 for 1 h. Cells were then washed twice with DMEM and cultured in infection medium (DMEM supplemented with 1 μg/mL TPCK-treated trypsin [T1426, Sigma-Aldrich, St. Louis, MO, USA]). Supernatants were collected at indicated times post-infection for viral titer determination.

### 2.9. Viral Binding and Entry Assay

HEK293T and A549 cells stably expressing empty vector (Flag) or Flag-CTSC were seeded in 6-well plates and cultured to appropriate density. Cells were incubated with H1N1 virus (MOI = 5) at 4 °C for 1 h to allow viral attachment without entry. Unbound viruses were removed by two washes with ice-cold PBS. Pre-warmed DMEM was then added, and cells were shifted to 37 °C for 30 min to permit viral internalization. After treatment with neuraminidase (Merck: N2876, Sigma-Aldrich, USA) at 37 °C for 30 min to remove surface-attached but non-internalized virions, cells were washed three times with PBS. Total cellular RNA was extracted, and viral *NP* gene copies were quantified by qRT-PCR. Relative copy numbers were calculated using the empty vector control group as a reference. All experiments were performed in triplicate with three independent repeats.

### 2.10. Histopathological Analyses

Mouse lung tissues were excised and fixed in 10% neutral buffered formalin, followed by dehydration and paraffin embedding. Tissue sections were cut longitudinally at a thickness of 4 µm. The slides were baked at 65 °C for 30–60 min, deparaffinized in xylene, and rehydrated through a graded ethanol series to distilled water before staining with H&E. Histopathological assessment was performed in a blinded manner by two board-certified pathologists. The following scoring criteria were applied: for edema, hemorrhage, hyaline membrane formation, and necrotic cellular debris—0, none; 1, rarely observed in <5% of lung fields (30× magnification); 2, present in up to 33% of lung fields; 3, present in 33–66% of lung fields; and 4, present in > 66% of lung fields. Infiltration of mononuclear cells and eosinophils was scored as: 0, within normal limits; 1, small aggregates in peribronchial and perivascular regions; 2, perivascular and peribronchial aggregates filling the perivascular space; and 3, extensive sheet-like infiltrates extending into septa and associated with consolidated lesions in lung regions. The overall severity of lung injury was graded according to a previously described 5-point scoring system.

### 2.11. Plaque Assay

Viral titers were determined by plaque assay. MDCK cell monolayers in 12-well plates were incubated with 0.25 mL of serial 10-fold dilutions of viral supernatants at room temperature (RT) for 1 h with gentle rocking every 15 min. Cells were then overlaid with 1 mL of 1% agarose containing 0.25% FBS. After solidification at RT, plates were inverted and incubated at 37 °C. At 72 h post-infection, agarose overlays were removed, and plaques were visualized by staining with 0.1% crystal violet. Viral titers were calculated and expressed as plaque-forming units (PFU) per mL.

### 2.12. Flow Cytometry Analysis

HEK293T and A549 cells were infected with PR8 tagged with GFP for 1 day (MOI = 0.1). For flow cytometry, GFP intensity was measured using an Attune NxT Acoustic Focusing Cytometer (Invitrogen, Carlsbad, CA, USA) with the FITC channel. Uninfected cells were used to set the voltage. Data were acquired with Attune NxT Software (v5.3) and analyzed using FlowJo (v10.8.1).

### 2.13. Western Blot

HEK293T and A549 Cells were infected with the H1N1 strains at 0.1 MOI for specified time. Cells were washed with cold PBS and lysed in RIPA Lysis Buffer (Strong) (CW2333S, CWBIO, Beijing, China) supplemented with protease and phosphatase inhibitor cocktails (P1006, P1081, Beyotime, China) for 30 min. Finally, the obtained cell supernatants were boiled at 100 °C for 10 min to obtain the cell proteins. Subsequent protein identification was performed via SDS-PAGE.

### 2.14. Quantitative Reverse Transcription Polymerase Chain Reaction (qRT-PCR) Assays

Total RNA was extracted from cultured cells using TRIzol reagent (Invitrogen, 15596026, Carlsbad, CA, USA), followed by reverse transcription with the 5× RTIII Mix (RR036A, Takara, Japan) according to the manufacturer’s protocol. Quantitative real-time PCR was performed using 2×KAPA SYBR FAST Universal qPCR Kit (KK4601, KAPA Biosystems, Wilmington, MA, USA) on the Applied Biosystems^®^ QuantStudio™ 6 Flex Real-Time PCR System (Applied Biosystems, Waltham, MA, USA). Relative gene expression was calculated via the 2^−ΔΔCt^ method, with human *ACTB* serving as the internal control. The real-time PCR cycling protocol was as follows: 95 °C for 30 s, followed by 40 cycles at 95 °C for 10 s and 60 °C for 30 s. All primer pairs were designed with Primer5 and are listed in Supplementary Table S2.

### 2.15. Statistical Analysis

Statistical analyses were performed using GraphPad Prism (v9.5). Data are presented as mean ± SD from at least three independent experiments. Comparisons between two groups were made using two-tailed unpaired Student’s *t* test unless otherwise indicated. Significance levels are denoted as * *p* < 0.05, ** *p* < 0.01, *** *p* < 0.001, **** *p* < 0.0001, n.s., not significant.

## 3. Results

### 3.1. CTSC Is Identified as an Early-Response Host Factor to Influenza Infection

**Figure 1:**
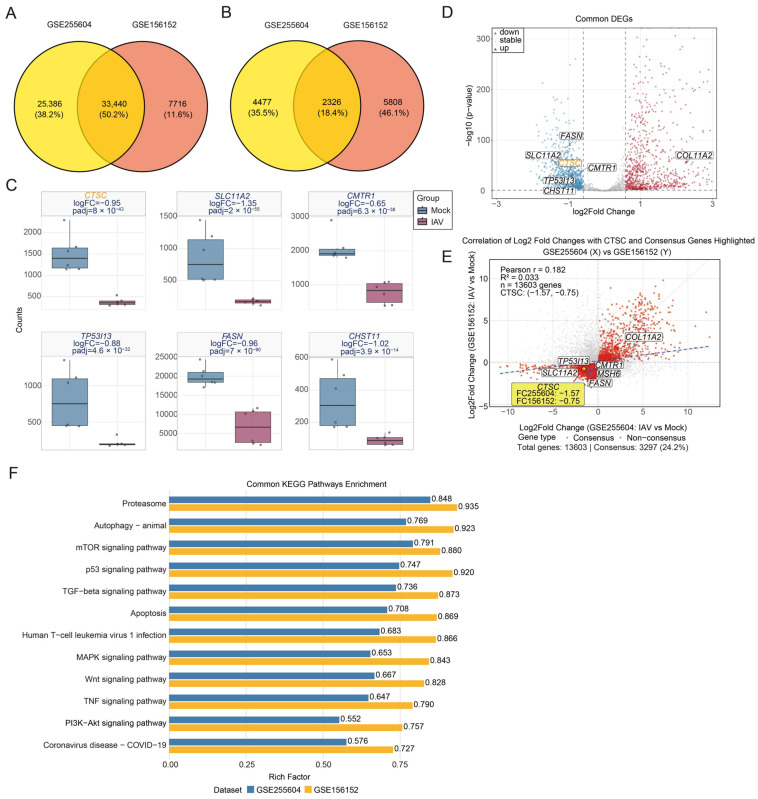
Integrative transcriptomic analysis identifies CTSC as an early downregulated host factor during influenza infection. (**A**) Venn diagram of overlapping genes between the GSE156152 and GSE255604 datasets. (**B**) Intersection of all differentially expressed genes (DEGs) from the GSE156152 and GSE255604 datasets. (**C**) Co-expression profiles of SLC11A2, CMTR1, TP53I13, FASN, CHST11, and CTSC across the two gene sets. (**D**) Volcano plot showing significantly upregulated and downregulated genes. (**E**) Correlation analysis illustrating expression patterns of common differentially expressed genes. (**F**) Shared KEGG enrichment pathways identified between the two gene sets. All experiments were conducted with at least three independent biological replicates. Representative results from one experiment are shown, and similar findings were obtained across all replicates.

To comprehensively dissect the genome-wide transcriptional regulatory network of host cells in response to influenza virus infection, we employed an integrative systems biology approach by analyzing two independent high-throughput transcriptomic datasets from the Gene Expression Omnibus (GEO): GSE156152 (BioProject: PRJNA656848, Human embryonic kidney 293T cells infected with the Wisconsin Swine (WSN) strain) and GSE255604 (Human lung epithelial A549 cells infected with the H1N1 strain). These complementary experimental systems model distinct yet relevant cellular targets of influenza infection, providing a comparative framework to identify conserved host transcriptional response mechanisms through systematic analysis of infected induced changes. Cross-dataset integration identified 33,440 common high-quality genes (Figure 1A). Using the DESeq2 algorithm on this integrated gene set, we performed differential expression analysis, which revealed a substantial number of significantly altered genes in each dataset. Venn diagram analysis demonstrated that 2326 genes were differentially expressed in both models (Figure 1B), representing a core set of genes likely reflective of a conserved host transcriptional response to influenza virus infection. To quantify the effect size of these shared differentially expressed genes while accounting for cross-experimental variability, we performed a meta-analysis using a random-effects model [21]. The distribution of pooled effect sizes f exhibited a typical bell-shaped curve (Supplementary Figure S1A), reflecting high data quality and stable effect estimates. Furthermore, a forest plot corroborated the significant downregulation of the lysosomal cysteine protease CTSC in both datasets (Supplementary Figure S1B).

Gene co-expression profiling of the integrated gene set revealed several distinct transcriptional modules. Among these, we identified multiple known influenza-associated genes, including the iron transporter *SLC11A2* (which regulates iron metabolism required for viral replication) [22], the methyltransferase *CMTR1* (involved in viral mRNA processing) [23], the stress-response gene *TP53I13*, the lipid metabolism enzyme *FASN* (supplying membrane components for viral assembly) [24], and the sulfation modifier *CHST11* (affecting viral receptor binding) [25]. In contrast, *CTSC* exhibited a consistent and pronounced down-regulation trend in both infection models (Figure 1C). Combined analysis further supported these findings, with a volcano plot demonstrating significant downregulation of these genes (Figure 1D). To validate the integrated analysis, we inspected the expression of these genes in each dataset individually and obtained consistent results (Supplementary Figure S1C,D).

We assessed the global correlation of expression changes for all shared genes between the two experimental systems, obtaining a Pearson correlation coefficient of r = 0.182 (Figure 1E). This relatively low correlation likely reflects cell-type-specific responses as well as technical variations. Notably, CTSC showed consistent downregulation across both datasets (Figure 1E). The distribution of gene counts across quadrants is provided in Supplementary Figure S1E. To investigate the biological implications of CTSC downregulation, we performed independent KEGG pathway enrichment analyses on each dataset. Although the overall transcriptional profiles differed between the two models, several key pathways were commonly enriched (Figure 1F and Supplementary Figure S1F). Among these pathways, proteasome and autophagy pathways were synchronously activated, both critically involved in protein degradation and cellular homeostasis. Moreover, the PI3K-AKT signaling pathway, a central regulator of cell survival and metabolism, was markedly activated in both infection models. Collectively, our integrated analysis reveals that the lysosomal cysteine protease CTSC is significantly and consistently downregulated across influenza virus infection models in two distinct cell types. This conserved expression pattern, together with coordinated activation of proteasome and autophagy pathways, suggests that CTSC may play an important and previously underappreciated role within the regulatory network governing influenza virus–host interactions. Furthermore, qRT-PCR analysis further validated the downregulation of *CTSC* mRNA levels in both 293T and A549 cells upon H1N1 infection (Supplementary Figure S1G,H).

### 3.2. CTSC Inhibits H1N1 Replication in Human Embryonic Kidney (HEK293T) and Respiratory Epithelial (A549) Cells

**Figure 2:**
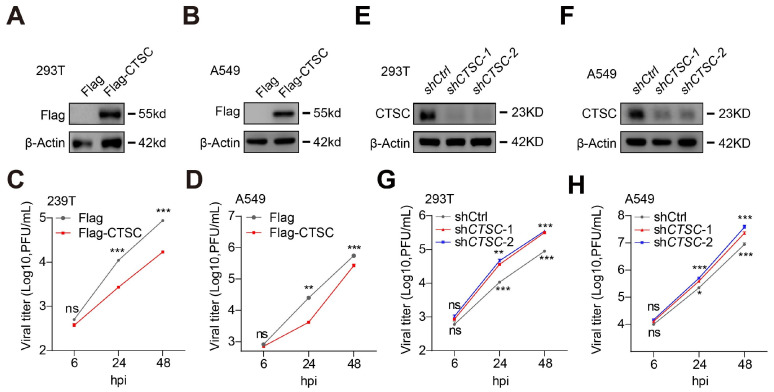
CTSC restrains H1N1 replication in vitro. (**A**) HEK293T-Flag and HEK293T-Flag-CTSC cells were constructed, and CTSC expression was confirmed by Western blotting. (**B**) A549-Flag and A549-Flag-CTSC cells were constructed and analyzed for CTSC expression by Western blotting. (**C**) The indicated cells (HEK293T-Flag and HEK293T-Flag-CTSC cells) were infected with H1N1-PR8 virus (MOI = 0.1). Viral titers in supernatants were quantified by plaque assay and expressed as plaque-forming units (PFU) per mL. (**D**) A549-Flag and A549-Flag-CTSC cells were infected with H1N1-PR8 (MOI = 0.1), and viral titers in supernatants were quantified by plaque assay at the indicated time points. (**E**) HEK293T cells were transfected with shRNA targeting CTSC, and knockdown efficiency was validated by Western blotting. (**F**) A549 cells were transfected with shRNA targeting CTSC, followed by CTSC expression determined via Western blotting. (**G**) CTSC-knockdown HEK293T cells were infected with H1N1-PR8 virus (MOI = 0.1). Viral titers in supernatants were determined by plaque assay. (**H**) CTSC-knockdown A549 cells were infected with H1N1 virus (MOI = 0.1) for the indicated times, and viral titers in supernatants were quantified by plaque assay. Data are expressed as mean ± SD of three independent biological replicates. Differences between groups were evaluated using two-tailed unpaired Student’s *t* test. *P* values less than 0.05 were considered statistically significant (* *p* < 0.05, ** *p* < 0.01, *** *p* < 0.001, n.s., not significant). No adjustments were made for multiple comparisons.

To investigate whether CTSC affects H1N1 replication, we performed gain- and loss-of-function experiments in human embryonic kidney (HEK293T) and respiratory epithelial (A549) cells. Firstly, we established HEK293T and A549 cell lines stably overexpressing CTSC (Figure 2A,B and Supplementary Figure S2A,B). These cells, along with their respective controls, were infected with H1N1 at low multiplicities of infection (MOI = 0.1), independently. Overexpression of CTSC significantly reduced progeny virus titers at 24 and 48 h post-infection in both cell types (Figure 2C,D). We next used shRNAs to knock down the endogenous CTSC in HEK293T and A549 cells (Figure 2E,F and Supplementary Figure S2C,D). Knockdown of CTSC resulted in significantly enhanced H1N1 replication compared with control cells (Figure 2G,H). In addition, the efficiency of CTSC overexpression and knockdown was confirmed by quantitative real-time (qRT-PCR) (Supplementary Figure S2E–H). Taken together, these results demonstrate that endogenous CTSC acts as a potent inhibitor of H1N1 replication in both HEK293T and A549 cells.

### 3.3. CTSC Inhibits the Expression of NP Protein of H1N1

**Figure 3:**
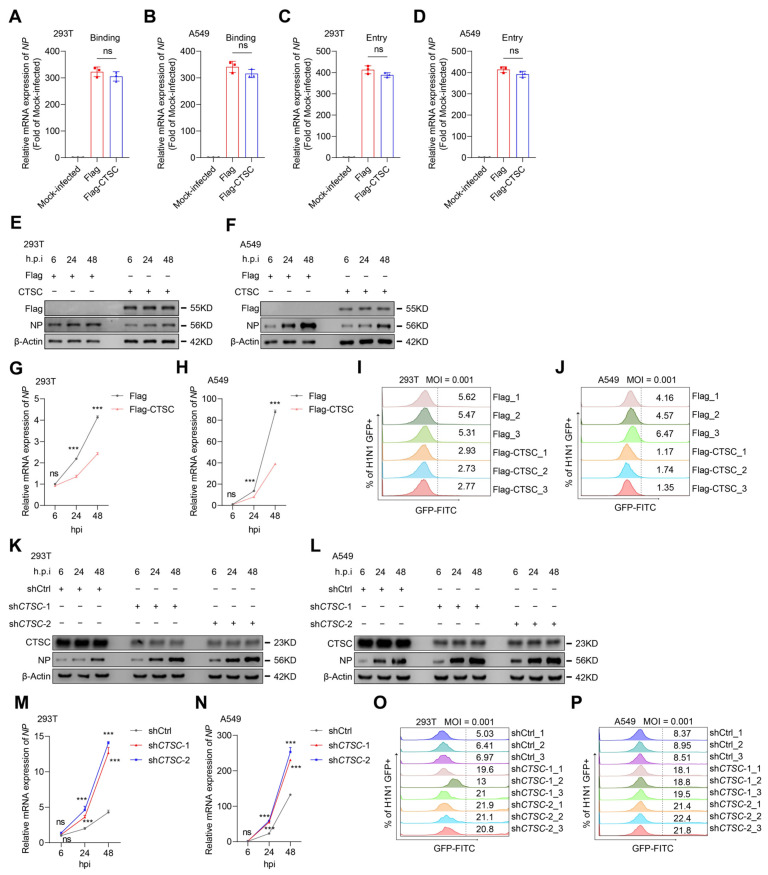
CTSC suppresses NP expression of H1N1 and does not affect viral binding or entry. (**A**–**D**) CTSC overexpression does not affect H1N1 binding or entry. HEK293T and A549 cells stably expressing empty vector or Flag-CTSC were infected with H1N1 at an MOI of 5. Viral NP gene copies were quantified by qRT-PCR. (**A**) Binding assay in HEK293T cells; (**B**) Binding assay in A549 cells; (**C**) Entry assay in HEK293T cells; (**D**) Entry assay in A549 cells. No significant difference was observed between the Flag-CTSC group and the empty vector control group. (**E**–**H**) Effects of CTSC overexpression on H1N1 NP protein and mRNA levels in HEK293T and A549 cells. Cells were harvested at 6, 24 and 48 h post-infection (MOI = 0.1). NP protein was detected by Western blotting, and NP mRNA was quantified by quantitative real-time PCR following RNA extraction using the TRIzol method. (**I**,**J**) GFP-labeled H1N1 virus was used to infect CTSC-overexpressed HEK293T and A549 cells for 24 h (MOI = 0.1). Viral replication levels were quantified by flow cytometry. (**K**–**N**) Effects of CTSC knockdown on H1N1 NP protein and mRNA levels in HEK293T and A549 cells. Protein and mRNA expression were analyzed at 6, 24, and 48 hpi (MOI = 0.1). (**O**,**P**) GFP-labeled H1N1 was used to infect CTSC-knockdown HEK293T and A549 cells for 24 h (MOI = 0.1). Viral replication was quantified by flow cytometry. Data are expressed as mean ± SD. Statistical analysis was performed using unpaired two-tailed Student’s *t* test (*** *p* < 0.001, n.s., not significant), no adjustments were made for multiple comparisons.

To investigate the role of CTSC in the viral life cycle, we first examined whether CTSC affects virus binding or entry. Cells stably expressing empty vector or Flag-CTSC were incubated with H1N1 virus for 1 h at 4 °C to allow virus binding, followed by incubation with pre-warmed medium at 37 °C to permit virus entry. Quantitative RT-PCR data showed that CTSC overexpression did not affect virus binding or entry (Figure 3A–D). Since CTSC did not affect viral entry, we next examined whether CTSC acts at a post-entry stage of viral replication. To this end, we examined the expression of the nucleoprotein (NP) of H1N1 at multiple time points post-infection in HEK293T and A549 cells. In cells overexpressing CTSC and infected with H1N1 virus (MOI = 0.1), both the protein and mRNA levels of NP were significantly reduced at 24 and 48 h post-infection compared with controls (Figure 3E–H and Supplementary Figure S3A,B). In addition, using a GFP-tagged PR8 virus, flow cytometry revealed a clear decrease in GFP-positive signal in CTSC-overexpressed HEK293T and A549 cells (Figure 3I,J). Conversely, shRNA-mediated knockdown of CTSC in HEK293T and A549 cells led to markedly increased protein and mRNA expression levels of NP (Figure 3K–N and Supplementary Figure S3C,D), which was corroborated by enhanced GFP signal in flow cytometry analyses (Figure 3O,P).

Taken together, these results demonstrate that CTSC does not affect H1N1 binding or entry but restricts viral infection by suppressing post-entry stages of viral replication, such as NP protein expression.

### 3.4. CTSC Suppresses H1N1 Replication In Vivo

**Figure 4:**
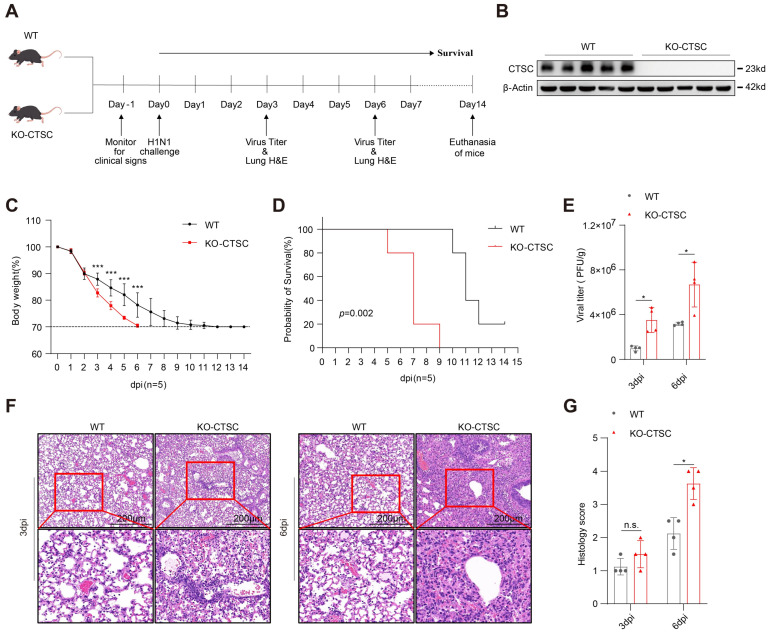
CTSC reduces H1N1 replication in vivo. Six-week-old male wild-type (WT) and *Ctsc*-knockout (*Ctsc*-KO) mice were physically examined one day before H1N1 infection. On day 0, fifteen mice per group were intranasally inoculated with 200 PFU of H1N1. Five mice per group were euthanized on days 3 and 6 post-infection. Lungs were homogenized for virus titer determination and processed for histopathology analysis. The remaining five mice per group were monitored for survival until day 14 post-infection. (**A**) Schematic overview of the experimental design. (**B**) CTSC protein expression in WT and *Ctsc*-KO mice were analyzed by Western blotting. (**C**) Body weight loss of mice (*n* = 5) over 14 days post infection. Mice losing > 30% of initial body weight were euthanized in accordance with animal welfare guidelines. (**D**) Survival curve of H1N1-infected mice (*n* = 5). (**E**) Lung virus titer on days 3 and 6 post infection (*n* = 4). (**F**) H&E-stained lung sections collected on day 3 and 6 post-infection. Scale bar: 200 μm. (**G**) Histological scores are presented as mean ± SD (*n* = 4). All experiments were performed with at least three independent biological replicates. Data are presented as mean ± SD. Statistical comparisons between two groups were performed using two-tailed unpaired Student’s *t* test. Significance levels are denoted as * *p* < 0.05, *** *p* < 0.001, n.s., not significant. No adjustments were made for multiple comparisons.

To investigate the role of CTSC during H1N1 infection in vivo, we generated *Ctsc*-specific knockout mice using CRISPR/Cas9 technology (Supplementary Figure S4A,B) and subsequently evaluated the impact of *Ctsc* deficiency on H1N1 infection and viral pathogenesis. Prior to infection, mouse genotypes were confirmed by PCR (Supplementary Figure S4C). 6-week-old wild-type (WT) and *Ctsc*-knockout (*Ctsc*-KO) mice were administered 200 PFU of PR8 virus intranasally and the CTSC knockout effect in lung was confirmed by Western blotting analyses (Figure 4A,B). Following infection, body weight and survival were monitored daily for 14 days. Mice in the *Ctsc* knockout group exhibited lower body weight and shortened survival time compared to those in the WT group (Figure 4C,D). Moreover, higher viral titers of H1N1 were detected in the lungs of *Ctsc*-knockout mice on day 3 and day 6 post-infection relative to the WT group (Figure 4E). In addition, Histological analyses of lung sections further revealed that *Ctsc*-knockout mice exhibited more severe lung injury than WT controls, characterized by enhanced inflammatory cell infiltration, thickened alveolar walls, and increased hemorrhage and congestion [26] (Figure 4F,G). Taken together, these findings suggest that *Ctsc* plays an important protective role against H1N1 infection and alleviates H1N1-induced pathogenicity in mice.

### 3.5. Transcriptomic Analysis Reveals a Significant Negative Correlation Between CTSC Expression and PI3K-AKT Pathway Activity

**Figure 5:**
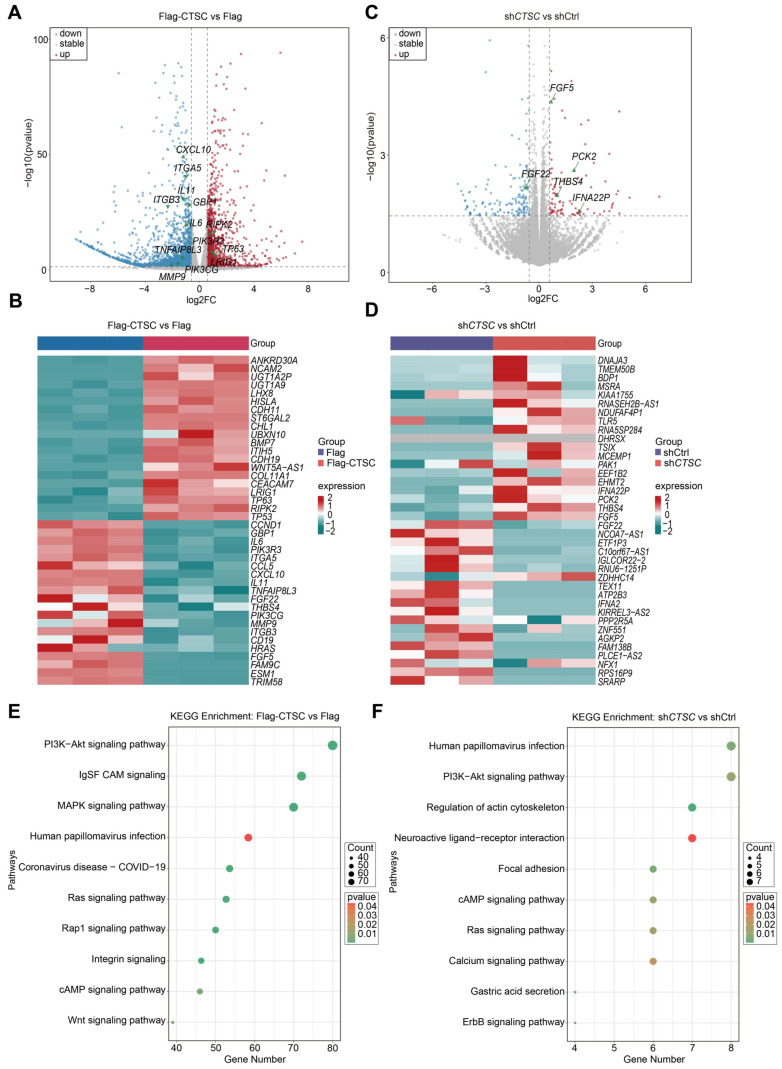
RNA-seq analysis reveals a negative association between CTSC expression and PI3K-AKT pathway activity. A549 cells with CTSC overexpressed or knockdown were infected with H1N1 (MOI = 0.1). Total RNA was extracted from cell pellets and subjected to RNA-seq analysis. Subsequent bioinformatic analyses were conducted using R Studio (v2025.09.2+418). (**A**) Volcano plot showing differentially expressed genes in CTSC-overexpressing (Flag-CTSC) versus control (Flag) A549 cells. (**B**) Heatmap depicting distinct clusters of significantly up- and down-regulated genes in CTSC-overexpressing versus control cells. (**C**) Volcano plot showing differentially expressed genes in CTSC-knockdown (sh*CTSC*) versus control (shCtrl) A549 cells. (**D**) Heatmap depicting distinct clusters of significantly up- and down-regulated genes in CTSC-knockdown versus control cells. (**E**,**F**) KEGG pathway enrichment analysis highlighting significant enrichment of the PI3K-AKT signaling pathway. All experiments were conducted with at least three biological replicates, and representative results are shown.

To further explore the mechanism by which CTSC exerts its anti-H1N1 effect, we performed RNA-sequencing analyses in A549 cells upon overexpression or knockdown of CTSC, infected with H1N1 at an MOI of 0.1 (Supplementary Figure S5A). In CTSC-overexpressed cells, genes associated with inhibition of the PI3K-AKT pathway (e.g., *RIPK2* and *LRIG1*) and pro-apoptotic genes (e.g., *TP63* and *TP53*) were significantly upregulated, whereas inflammatory factors (e.g., *CXCL10*, *IL6*, *IL11* and *TNF)* were significantly downregulated (Figure 5A,B). Conversely, CTSC-knockdown cells showed upregulation of genes linked to PI3K-AKT activation (e.g., *THBS4*, *FGF5* and *PCK2*) and elevated expression of *TNF* (Figure 5C,D). GO enrichment analysis revealed significant enrichment in biological processes related to lung respiratory system development, growth factor binding, fibroblast growth factor receptor signaling pathway, innate immunity, and viral genome replication (Supplementary Figure S5B–E). Meanwhile, KEGG pathway analysis further highlighted prominent enrichment of the PI3K-AKT signaling pathway (Figure 5E,F). GSEA results demonstrated that inflammation-related pathways were significantly downregulated in the CTSC-overexpressed group but upregulated in CTSC-knockdown cells (Supplementary Figure S5F–K). Taken together, integrated transcriptomic and bioinformatic analyses consistently underscore negative correlation between CTSC expression and PI3K-AKT pathway activity, suggesting its potential functional importance in host-virus interactions during influenza infection.

### 3.6. CTSC Attenuates the PI3K-AKT Pathway

**Figure 6:**
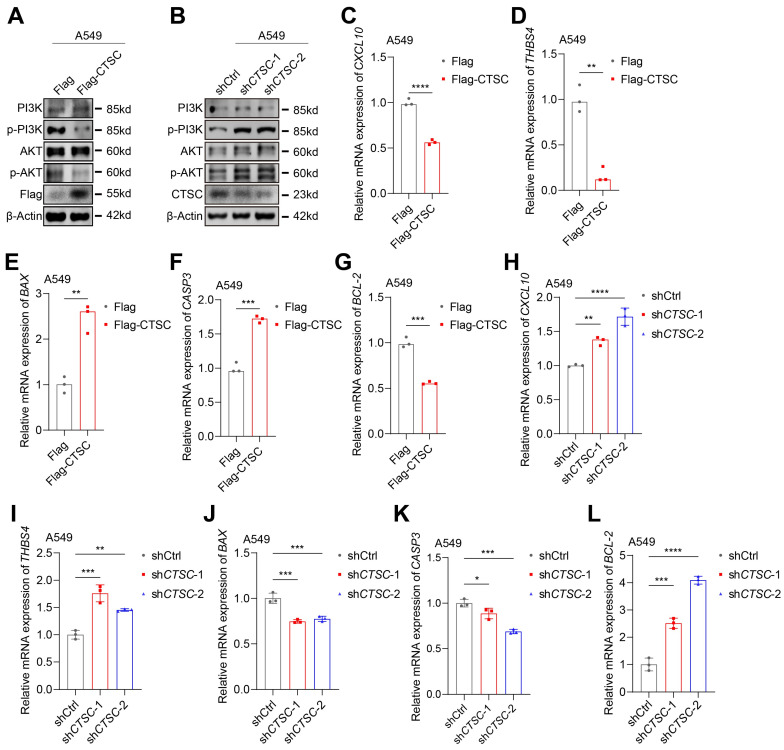
CTSC downregulates H1N1-activated PI3K-AKT signaling pathway. (**A**) Following CTSC overexpression, phosphorylation levels of PI3K (p-PI3K) and AKT (p-AKT) were reduced upon CTSC overexpression in A549 cells. (**B**) CTSC knockdown increased p-PI3K and p-AKT expression in A549 cells. (**C**,**D**) Expression of PI3K-AKT pathway-related genes, *CXCL10* and *THBS4* were markedly downregulated in CTSC-overexpressing A549 cells. (**E**–**G**) Pro-apoptosis-associated genes, *BAX*, *CASP3* were markedly upregulated in CTSC-overexpressing A549 cells (**E**,**F**). Anti-apoptosis-associated genes, *BCL-2* was markedly downregulated in CTSC-overexpressing A549 cells (**G**). (**H**–**L**) *CXCL10* and *THBS4* were markedly upregulated in A549 knockdown cells (**H**,**I**). *BAX*, *CASP3* were markedly downregulated in A549 knockdown cells (**J**,**K**). *BCL-2* was markedly upregulated in A549 knockdown cells (**L**). The data are presented as means ± SD from three biologically independent experiments (n = 3). Statistical significance was assessed using an unpaired, two-tailed Student’s *t* test. (* *p* < 0.05; ** *p* < 0.01; *** *p* < 0.001, **** *p* < 0.0001).

To investigate the molecular mechanism by which CTSC restricts IAV replication, we next examined its effect on the PI3K-AKT pathway, a critical host axis known to promote viral replication and cell survival. A549 cells with stable CTSC overexpression or knockdown were infected with H1N1 (MOI = 0.1) for 24 h, and the activation status of the pathway was assessed. Western blot analysis revealed that CTSC overexpression led to a significant downregulation of phosphorylated PI3K (p-PI3K) and AKT (p-AKT) (Figure 6A, Supplementary Figure S6A,B). Conversely, CTSC knockdown resulted in a marked upregulation of these activated forms (Figure 6B, Supplementary Figure S6C,D). Consistently, qRT-PCR analysis showed that CTSC overexpression significantly reduced the mRNA levels of PI3K-AKT pathway-associated genes, such as CXCL10 and THBS4 (Figure 6C,D,H,I) [27,28]. These results demonstrate that CTSC acts as a negative regulator of the PI3K-AKT signaling axis during IAV infection.

To determine whether the observed changes in the PI3K-AKT pathway-related genes (*CXCL10* and *THBS4*) are driven by viral infection, we examined their expression under mock-infected conditions. As shown in Supplementary Figure S7A–D, neither CTSC overexpression nor knockdown significantly altered the expression of these genes in the absence of viral infection. These results indicate that the regulatory effects of CTSC on the PI3K-AKT pathway occur specifically in the context of viral infection.

### 3.7. The PI3K-AKT Pathway Is a Critical Pro-Viral and Anti-Apoptotic Hub in IAV Infection

The PI3K-AKT signaling pathway is well-documented for its classic anti-apoptotic functions, which are frequently hijacked by viruses to promote replication. During IAV infection, the viral NS1 protein directly binds to the p85 regulatory subunit of PI3K, activating the pathway [29]. This activation leads to the phosphorylation and inhibition of downstream pro-apoptotic molecules such as caspase-9 and GSK-3β, thereby limiting virus-induced cell death and ensuring efficient viral replication. Further research has revealed that PI3K-AKT activation also negatively regulates the JNK pathway via ASK1, inhibiting Bax-mediated mitochondrial apoptosis [30].

Beyond its direct role in cell survival, the PI3K-AKT pathway is also a key orchestrator of the host inflammatory response. Inhibition of PI3K-AKT activation has been shown not only to induce apoptosis in infected cells but also to attenuate excessive host inflammatory responses [31]. Supporting this, a recent study demonstrated that pharmacological inhibition of the PI3K-AKT pathway significantly downregulates IAV-induced pro-inflammatory cytokines (IL-6, TNF-α) and interferons (IFN-β, ISG15), indicating that pathway activation is necessary for the virus-induced cytokine storm [31].

Thus, by hijacking the PI3K-AKT pathway, IAV achieves a dual benefit: it maintains host cell survival to support viral replication while simultaneously modulating inflammatory responses to enhance its pathogenicity. The pathway facilitates viral replication by suppressing premature apoptosis during the later stages of infection, thereby securing sufficient time for viral propagation. Consequently, when the PI3K-AKT pathway is inhibited, virus-induced apoptosis is significantly enhanced, and viral replication levels are reduced.

Given our finding that CTSC suppresses PI3K-AKT signaling activation, we next examined its impact on the expression of key apoptosis-related molecules. Notably, under mock-infected conditions, CTSC modulation did not affect the basal expression levels of *BAX*, *CASP3*, or *BCL-2* (Supplementary Figure S7E–J), indicating that the regulatory role of CTSC is infection-dependent. In infected cells, we assessed the expression of these factors. In line with the inhibitory effect of CTSC on the pro-survival PI3K-AKT pathway, CTSC overexpression significantly upregulated the pro-apoptotic genes *BAX* and *CASP3* while downregulating the anti-apoptotic gene *BCL-2* (Figure 6E–G). Conversely, CTSC knockdown produced the opposite expression pattern, suppressing pro-apoptotic genes and enhancing *BCL-2* expression (Figure 6J–L).

In addition, we assessed the expression of inflammatory factors and interferons in A549 cells upon overexpression or knockdown of CTSC. The results showed that, overexpression of CTSC significantly downregulated the levels of inflammatory factors (e.g., *IL6*, *TNF*, *STAT1*, *ISG15*, *GBP1* and *IL11*) and interferons (e.g., *IFNA1* and *IFNB1*), whereas knockdown of CTSC significantly upregulated the levels of these inflammatory factors and interferons, compared to the control group (Supplementary Figure S6E–T).

Taken together, these results demonstrate that CTSC functions as a key host restriction factor by attenuating PI3K-AKT-mediated pro-inflammatory and anti-apoptotic signaling, thereby limiting IAV replication and promoting a pro-apoptotic state in infected cells.

## 4. Discussion

**Figure 7:**
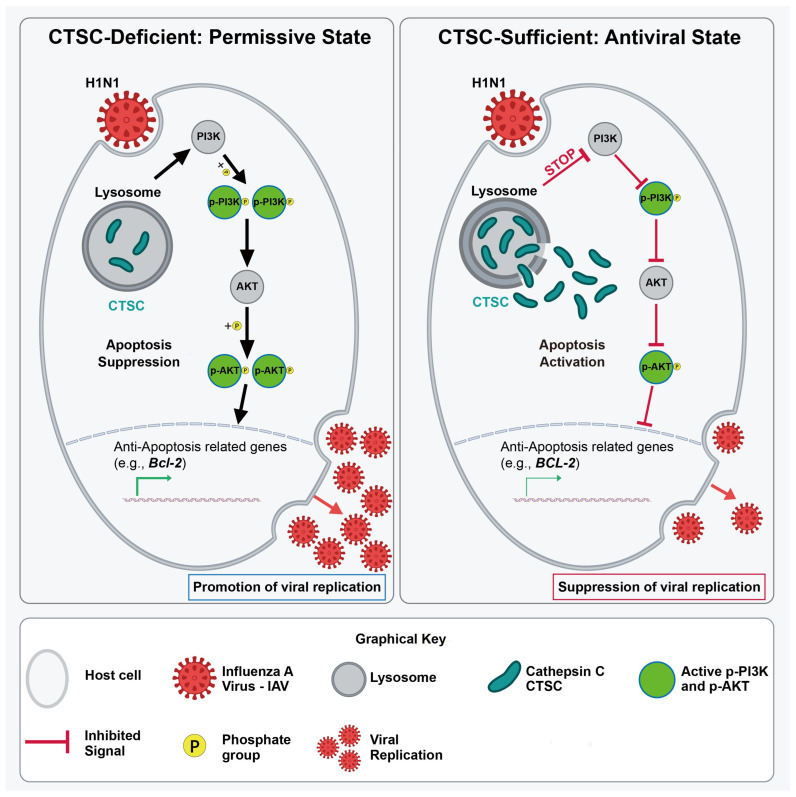
Proposed model for CTSC-mediated antiviral defense against influenza A virus via PI3K/AKT pathway modulation. (**Left**) In the absence of CTSC (CTSC-deficient permissive state), H1N1 infection triggers activation of PI3K, which is subsequently phosphorylated (p-PI3K). Activated p-PI3K phosphorylates AKT to generate p-AKT, which translocates to the nucleus and upregulates anti-apoptotic gene expression (e.g., *Bcl-2*). This suppression of apoptosis creates a cellular environment permissive for robust viral replication and progeny virus release. (**Right**) In CTSC-sufficient cells (antiviral state), CTSC expression blocks H1N1-induced PI3K activation (indicated by STOP symbol and red inhibitory lines), preventing downstream phosphorylation of PI3K and AKT. The resulting AKT inactivation leads to failure of anti-apoptotic gene induction, activation of apoptosis, and consequent suppression of viral replication.

The dynamic regulation of host factors during influenza virus infection critically determines viral pathogenicity and immune outcomes. By integrating transcriptomic data from multiple H1N1-infected models, we consistently identified CTSC as one of the most prominently downregulated host genes during early infection. Our findings revealed that CTSC-mediated inhibition of the PI3K-AKT signaling pathway plays a critical role in suppressing viral replication (Figure 7). This observation aligns with accumulating evidence that lysosomal proteases, including cathepsins, are actively involved in viral entry, replication, and immune regulation [32].

Functional experiments demonstrated that CTSC overexpression significantly reduced viral titers and NP expression, whereas CTSC knockdown or genetic deficiency exacerbated infection. Collectively, these in vitro and in vivo results establish CTSC as a host-restriction factor that limits efficient influenza virus replication, rather than merely serving as a post-infection inflammatory marker.

The role of cathepsin family members in viral infections is complex and context dependent. In contrast to our findings, cathepsin W (CTSW) has been reported to facilitate IAV replication by mediating viral escape from late endosomes, with CTSW-deficient mice showing 25% increased survival upon viral challenge [33,34]. Notably, the antiviral function of CTSC is evolutionarily conserved—from shrimp (*Fenneropenaeus chinensis*) (Fc-Cath C) to mammals. Studies have shown that CTSC from Chinese white shrimp is significantly upregulated upon viral challenge, whereas CTSC knockdown promotes viral replication [35], suggesting that the antiviral role of CTSC transcends species boundaries.

Beyond its evolutionary conservation, the antiviral function of CTSC is also mediated through distinct molecular mechanisms. A recent study by Chen et al. demonstrated that CTSC contributes to host defense against IAV by promoting the degradation of cortactin, an actin-binding protein that facilitates viral infection [36]. In that context, CTSC acts by directly cleaving a proviral host factor to restrict late-stage viral release. In contrast, our study identifies a signaling-based mechanism whereby CTSC attenuates the PI3K-AKT pathway to modulate apoptosis and inflammation. Together, these findings reveal that CTSC functions as a multifaceted antiviral factor, employing both proteolytic and signaling-regulatory mechanisms to restrict IAV infection. This mechanistic diversity underscores the central role of CTSC as a key coordinator of host antiviral immunity.

Mechanistically, we demonstrate that CTSC exerts its antiviral effects through inhibition of the PI3K-AKT signaling pathway—a central regulator of cell survival exploited by multiple viruses to sustain host cell viability [37]. We provide the first evidence linking CTSC downregulation to modulation of this pathway, proposing that CTSC upregulation suppresses viral replication by inhibiting PI3K-AKT signaling.

By suppressing PI3K-AKT, CTSC simultaneously modulates two processes co-opted by IAV to enhance replication and exacerbate immunopathology: apoptosis resistance and inflammatory responses. The PI3K-AKT pathway is well-documented to promote anti-apoptotic signaling, thereby providing a time window for viral replication by preventing premature host cell death [37]. Concurrently, emerging evidence demonstrates that PI3K-AKT activation is also involved in regulating inflammatory responses during viral infection. Thus, CTSC-mediated PI3K-AKT inhibition may simultaneously impair viral exploitation of anti-apoptotic mechanisms and modulate inflammatory responses. As a key activator of neutrophil serine proteases, the role of CTSC in inflammation regulation is well-established [38]. Our observation that early CTSC downregulation may promote viral replication through dual mechanisms—directly disinhibiting PI3K-AKT signaling and amplifying neutrophil-driven inflammation—positions CTSC as a regulatory hub coordinating viral replication and host inflammatory responses. Sustained or excessive inflammation is a hallmark of severe influenza and contributes to tissue injury [39], highlighting the therapeutic potential of pharmacologically modulating CTSC activity. Given that CTSC suppresses viral replication while also influencing inflammatory responses, fine-tuning rather than simply enhancing or inhibiting CTSC function may represent a promising host-directed strategy—balancing the dual imperative of antiviral defense and control of immunopathology.

Pathway enrichment analysis revealed PI3K-AKT as one of the most substantially suppressed pathways by CTSC. The PI3K-AKT pathway is shown to be exploited by multiple viruses to support their replication [40]. We hypothesize that CTSC, through its protease activity, may cleave and inactivate an upstream activator of the PI3K-AKT pathway, or alternatively, cleave and activate an upstream inhibitor—a possibility meriting future investigation through substrate-identification methods such as immunoprecipitation-mass spectrometry (IP-MS).

Several limitations warrant consideration. First, the direct molecular targets of CTSC protease activity that mediate PI3K-AKT suppression remain elusive. Second, the specific cell types mediating the protective effects of CTSC in vivo require precise definition. Third, the potential roles of CTSC in later stages (e.g., tissue repair) warrant further investigation. Finally, correlative studies in human cohorts are needed to establish translational relevance. Additionally, although our binding and entry assays demonstrate that CTSC does not affect viral binding or entry, the precise post-entry steps targeted by CTSC—whether viral RNA replication, protein translation, assembly, or egress—remain to be fully elucidated.

In summary, this study systematically demonstrates that CTSC is downregulated early during influenza virus infection and establishes its inhibitory role in viral replication through in vitro and in vivo models. Mechanistically, CTSC creates an intracellular environment unfavorable for viral replication by modulating the PI3K-AKT pathway. These findings advance our understanding of virus–host interactions and provide a rationale for developing CTSC-targeted host-directed anti-influenza strategies.

## 5. Conclusions

This study identifies CTSC as a host restriction factor that is downregulated during early influenza A virus infection and demonstrates that CTSC inhibits H1N1 replication both in vitro and in vivo. The antiviral function of CTSC is associated with suppression of the PI3K-AKT signaling pathway, leading to enhanced apoptosis and attenuated inflammatory responses. These findings establish CTSC as a key coordinator of host antiviral immunity and suggest that modulation of CTSC activity may represent a promising host-directed strategy for influenza treatment.

## Data Availability

The data that support the findings will be available in NCBI BioProject database at https://www.ncbi.nlm.nih.gov/bioproject/PRJNA1474100 (accessed on 30 May 2026), under accession number PRJNA1474100 following an embargo from the date of publication to allow for commercialization of research findings.

## Supplementary Materials

The following supporting information can be downloaded at: https://www.mdpi.com/article/10.3390/v18060641/s1, Figure S1: CTSC expression is significantly downregulated during influenza infection based on integrated analysis of public transcriptomic datasets; Figure S2: Validation of CTSC overexpression and knockdown in both constructed HEK293T and A549 cell lines; Figure S3: Impact of CTSC modulation on H1N1 infection; Figure S4: Generation and validation of *Ctsc* knockout mice; Figure S5: Transcriptomic profiling reveals significant enrichment of immune and inflammation pathways upon CTSC overexpression or knockdown; Figure S6: Analysis of host immune responses-related gene expression in CTSC-perturbed A549 cell lines; Figure S7. CTSC does not alter the expression of PI3K AKT pathway related or apoptosis related factors under mock infected conditions; Table S1: Sequences of gRNA and shRNA targeting CTSC; Table S2: Sequences of primer pairs used for qRT-PCR assays.

## Abbreviations

The following abbreviations are used in this manuscript:

CTSC	Cathepsin C
GEO	Gene Expression Omnibus
GSEA	Gene Set Enrichment Analysis
KEGG	Kyoto Encyclopedia of Genes and Genomes
GO	Gene Ontology
WT	Wild-Type
MOI	Multiplicity of Infection
MSigDB	Molecular Signatures Database
PFUs	Plaque-Forming Units
