# Supplementary material for: Cathepsin C Restricts Influenza A Virus Replication and Is Associated with Suppression of the PI3K-AKT Signaling Pathway
Source: Viruses. 2026 Jun 3;18(6):641. doi: 10.3390/v18060641 (PMC13307861; doi:10.3390/v18060641)
Supplement: Supplementary file 1 [file viruses-18-00641-s001.zip › viruses-4302364-supplementary/viruses-4302364-supplementary - edit/Supplementary materials/Supplementary materials.pdf]

Cathepsin C Restricts Influenza A Virus Replication and Is Associated with Suppression of the PI3K-AKT Signaling Pathway

Yansheng Zhu [1,†], Lanlan Si [2,†], Zhongzhong Cao [3], Huiyang Song [4,5], Aiqing Yang [4,5], Xia Wang [4,5], Yifei Qiu [4,5], Chengming Gao [4,5,‡], Gangqiao Zhou [1,2,3,4,5,*,‡] and Pengbo Cao [4,5,*]

[1] College of Life Sciences, Anhui Medical University, Hefei, 230000, China; yszhu810@163.com
[2] Graduate School, Guangzhou Medical University, Guangzhou, 511436, China; sll1900@163.com
[3] College of Life Sciences, Hebei University, Baoding, 071002, China; 19515660618@163.com
[4] Academy of Military Medical Sciences, Beijing, 100850, China; song_hickory@163.com (H.S.); yan_gaiqing_ok@126.com (A.Y.); 18589558796@163.com (X.W.); 3180101060@zju.edu.cn (Y.Q.); gchengming1988@163.com (C.G.)
[5] State Key Laboratory of Medical Proteomics, National Center for Protein Sciences at Beijing, Beijing, 100850, China
* Correspondence: zhougangqiao@ncpsb.org.cn (G.Z.); caopengbo@ncpsb.org.cn (P.C.); Tel.: +86-010-66931201 (G.Z.); +86-010-61777099 (P.C.)
† These authors contributed equally to this work.
‡ These authors also contributed equally to this work.

#Correspondence and address requesting for reprints to:

Dr. Pengbo Cao, State Key Laboratory of Medical Proteomics, National Center for Protein Sciences at Beijing, 27 Taiping Road, Beijing 100850, P.R. China. E-mail: caopengbo@ncpsb.org.cn; Phone: (86)-010-61777099.

OR

Dr. Gangqiao Zhou, State Key Laboratory of Medical Proteomics, National Center for Protein Sciences at Beijing, 27 Taiping Road, Beijing 100850, P.R. China. E-mail: zhougangqiao@ncpsb.org.cn; Phone & fax: (86)-010-66931201.

Index

Supplementary Figures

Supplementary Figure 1. CTSC expression is significantly downregulated during influenza infection based on integrated analysis of public transcriptomic datasets.

Supplementary Figure 2. Validation of CTSC overexpression and knockdown in both constructed HEK293T and A549 cell lines.

Supplementary Figure 3. Impact of CTSC modulation on H1N1 infection.

Supplementary Figure 4. Generation and validation of *Ctsc* knockout mice.

Supplementary Figure 5. Transcriptomic profiling reveals significant enrichment of immune and inflammation pathways upon CTSC overexpression or knockdown.

Supplementary Figure 6. Analysis of host immune responses-related gene expression in CTSC-perturbed A549 cell lines.

Supplementary Figure 7. CTSC does not alter the expression of PI3K-AKT pathway-related or apoptosis-related factors under mock-infected conditions.

Supplementary Figure 1

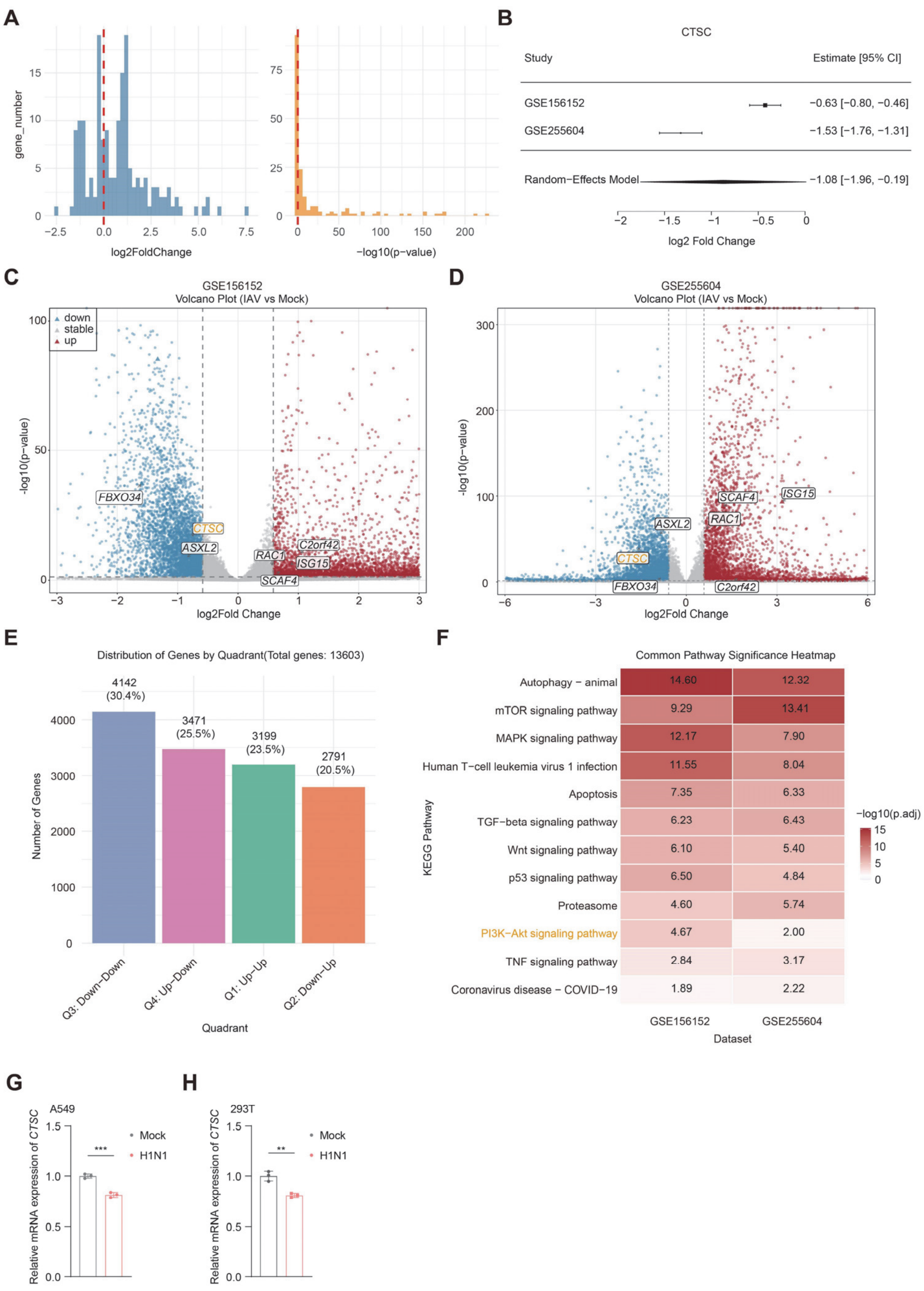

Supplementary Figure 1. CTSC expression is significantly downregulated during influenza infection based on integrated analysis of public transcriptomic datasets.

(A) The distribution of pooled effect sizes across all analyzed genes, approximating a normal (bell-shaped) curve. The position of *CTSC* is highlighted.

(B) Forest plot illustrating *CTSC* expression levels in the two gene sets.

(C, D) Volcano plot of differentially expressed genes in two independent datasets.

(E) The distribution of gene counts across quadrants is illustrated in the figure.

(F) Heatmap showing common enriched pathways between the two gene sets, with color intensity representing $-\log_{10}$ (adjusted *P*-value).

All experiments were conducted with biological replicates, and representative results are shown.

(G, H) qRT-PCR validation of CTSC downregulation upon H1N1 infection. 293T and A549 cells were infected with H1N1 (MOI = 0.1) for 24 h. *CTSC* mRNA levels were measured by qRT-PCR and normalized to mock-infected controls.

The data are presented as means ± SD from three independent experiments. Statistical analysis was performed using unpaired, two-tailed Student's *t*-test. (***P* < 0.01, ****P* < 0.001.).

Supplementary Figure 2

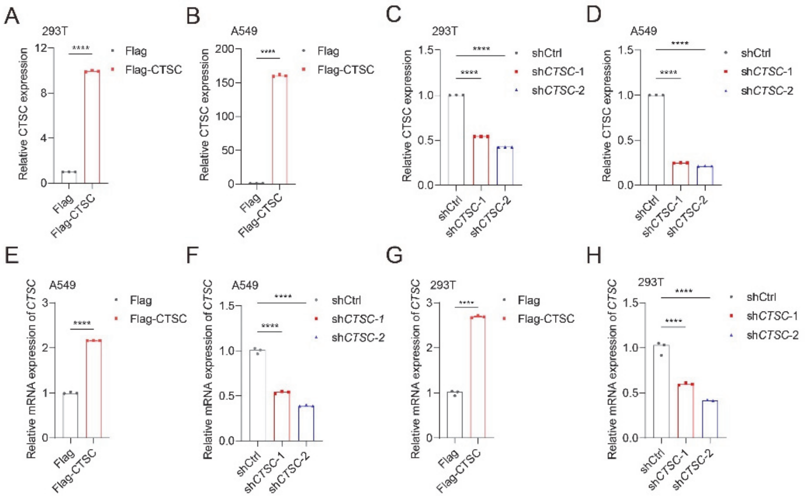

Supplementary Figure 2. Validation of CTSC overexpression and knockdown in both constructed HEK293T and A549 cell lines.

(A-D) Western blotting analysis confirming CTSC protein levels following overexpression (OE) or knockdown (KD) in HEK293T **(A, B)** and A549 **(C, D)** cells. **(E-H)** qRT-PCR analysis of *CTSC* mRNA levels following OE or KD in HEK293T **(E, F)** and A549 **(G, H)** cells.

The data are presented as means ± SD (n = 3 biologically independent experiments). Statistical analysis was performed using unpaired, two-tailed Student’s *t*-test. (*****P* < 0.0001).

Supplementary Figure 3

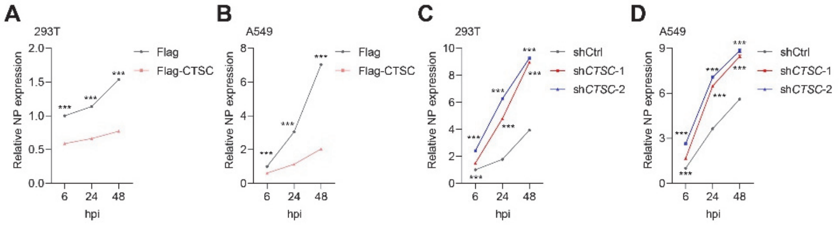

Supplementary Figure 3. Impact of CTSC modulation on H1N1 infection.

(A-D) Western blotting analysis confirmed NP protein expression in HEK293T and A549 cells at 6, 24 and 48 h post-infection with IAV (MOI = 0.1), following the OE or KD of CTSC.

The data are presented as means ± SD (n = 3 biologically independent experiments). Statistical analysis was performed using unpaired, two-tailed Student's *t*-test. (***$P <$ 0.001).

Supplementary Figure 4

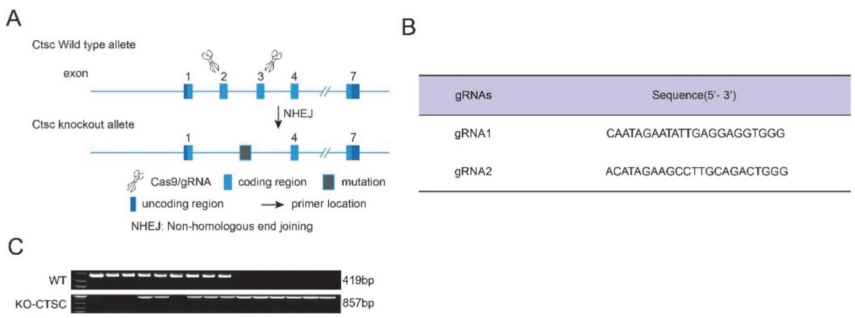

Supplementary Figure 4. Generation and validation of *Ctsc* knockout mice.

(A) Schematic illustrating the CRISPR/Cas9-mediated knockout strategy for the murine *Ctsc* gene.

(B) Sequences of the single-guide RNAs (sgRNAs) used to target *Ctsc*.

(C) Representative PCR genotyping results identifying wild-type (WT) and *Ctsc* knockout (KO) mice.

The experiment shown in panel C conducted with three biological replicates, and representative results are shown.

Supplementary Figure 5

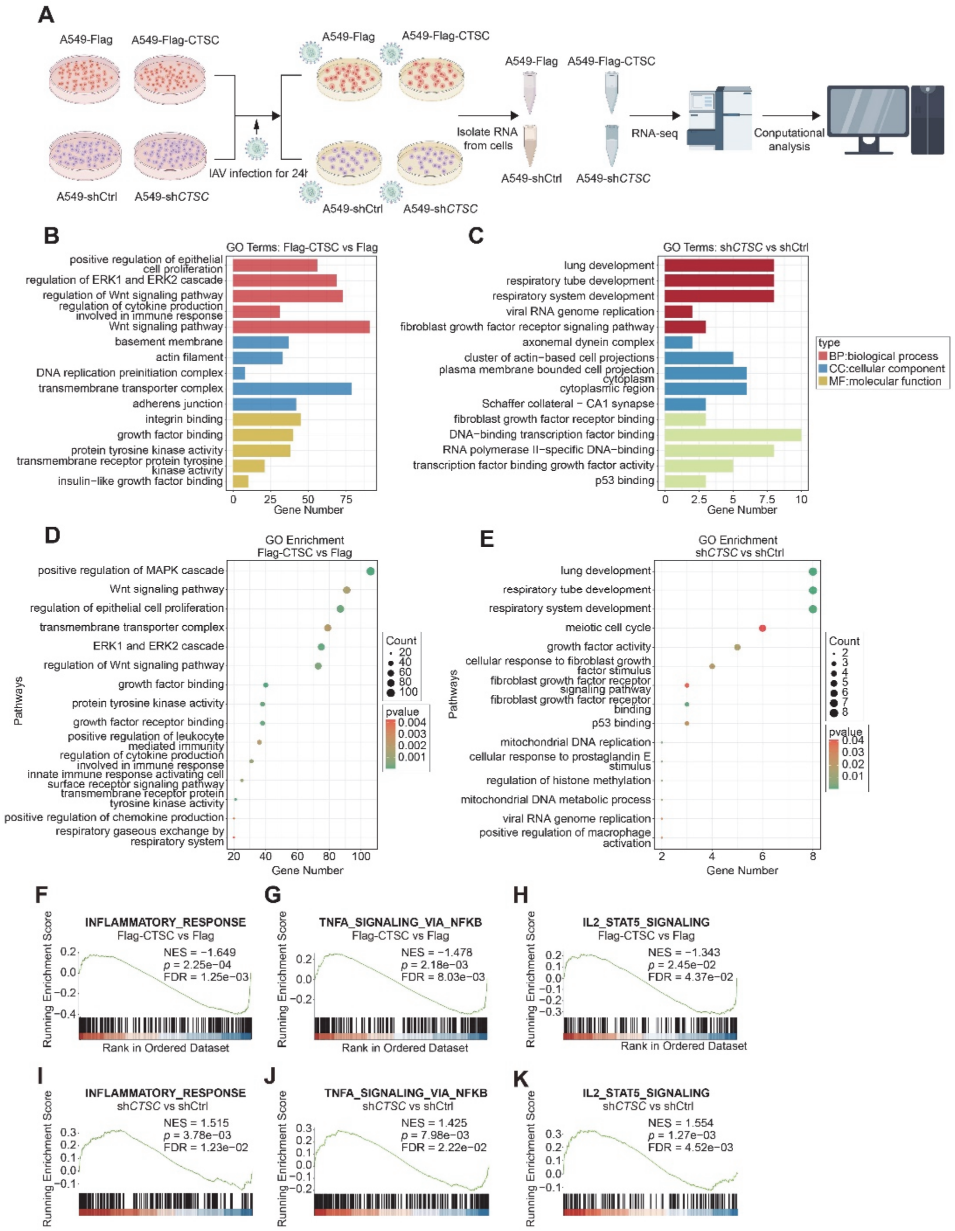

Supplementary Figure 5. Transcriptomic profiling reveals significant enrichment of immune and inflammation pathways upon CTSC modulation.

(A) Schematic overview of the RNA-sequencing and bioinformatic analysis workflow.

(B-E) Gene Ontology (GO) enrichment analysis revealed significant enrichment of pathways related to innate immune response and viral genome replication in CTSC-knockdown versus control cells.

(**F-H**) GSEA of RNA-seq data from CTSC-overexpressing (Flag-CTSC) versus control (Flag) A549 cells. Significant negative enrichment of inflammation-related

pathways was observed, including *Inflammatory response* (**F**), *TNFα signaling via NF-κB* (**G**), and *IL-2/STAT5 signaling* (**H**).

(**I-K**) GSEA of RNA-seq data from CTSC-knockdown (sh*CTSC*) versus control (shCtrl) A549 cells. In contrast, these pathways—*Inflammatory response* (**I**), *TNFα signaling via NF-κB* (**J**), and *IL-2/STAT5 signaling* (**K**)—showed significant positive enrichment in *CTSC*-knockdown cells. NES, normalized enrichment score.

All experiments were conducted with three biological replicates, and representative results are shown.

Supplementary Figure 6

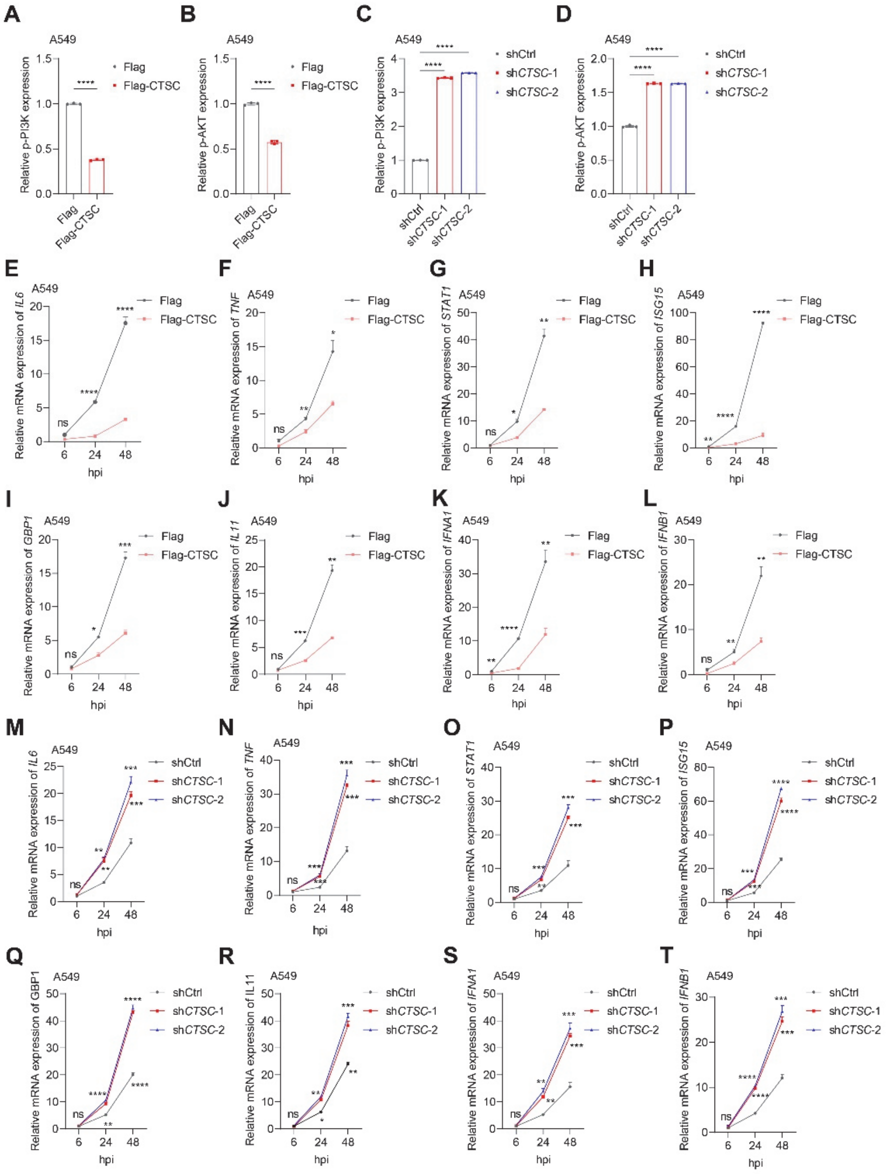

Supplementary Figure 6. Host immune responses in A549 cells.

(A–D) Densitometric quantification of PI3K-AKT pathway-related proteins. (**A**) Quantification of p-PI3K protein levels in the overexpression group; (**B**) Quantification of p-AKT protein levels in the overexpression group; (**C**) Quantification of p-PI3K protein levels in the knockdown group; (**D**) Quantification of p-AKT protein levels in the knockdown group.

(E–T) Effects of CTSC overexpression or knockdown on the expression of inflammatory factors and interferons in A549 cells. Overexpression group (**E–L**): (**E**)

IL6, (**F**) *TNF*, (**G**) *STAT1*, (**H**) *ISG15*, (**I**) *GBP1*, (**J**) *IL11*, (**K**) *IFNA1*, (**L**) *IFNB1*. CTSC overexpression significantly downregulated the expression levels of these factors. Knockdown group (**M–T**): (**M**) *IL6*, (**N**) *TNF*, (**O**) *STAT1*, (**P**) *ISG15*, (**Q**) *GBP1*, (**R**) *IL11*, (**S**) *IFNA1*, (**T**) *IFNB1*. CTSC knockdown significantly upregulated the expression levels of these factors. The data shown in panels are presented as means ± SD (n = 3 biologically independent experiments). Statistical analysis was performed using an unpaired, two-tailed Student's *t* test. (n.s, not significant, *$P < 0.05$, **$P < 0.01$, ***$P < 0.001$, ****$P < 0.0001$).

Supplementary Figure 7

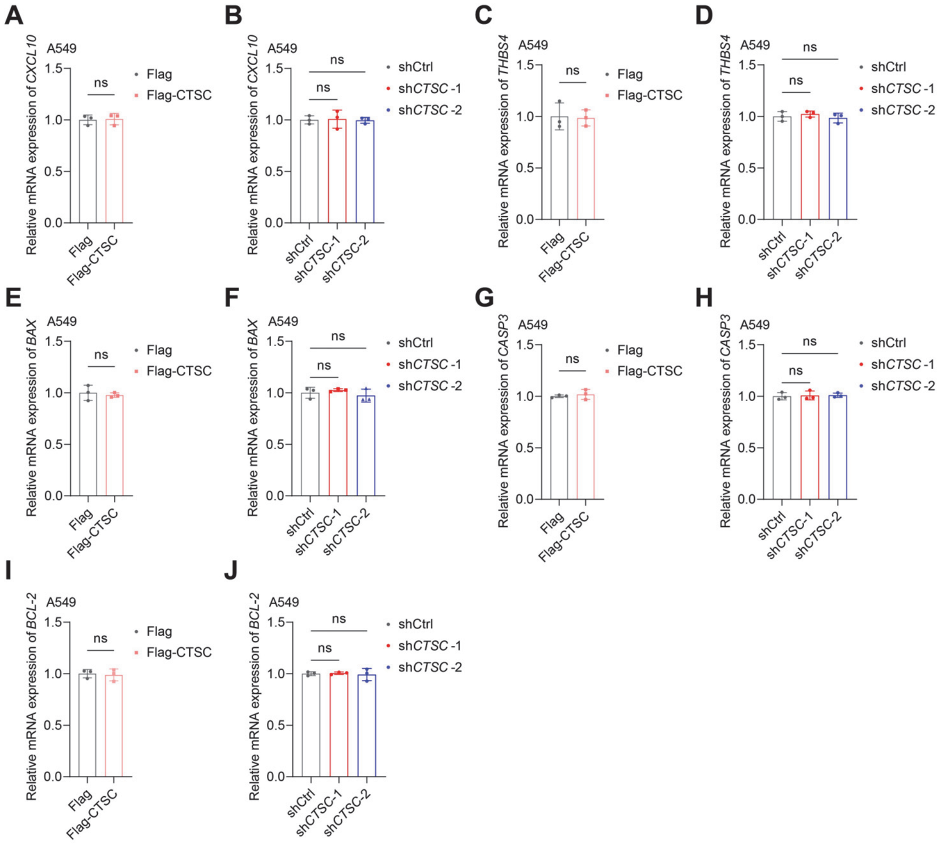

Supplementary Figure 7. CTSC does not alter the expression of PI3K-AKT pathway-related or apoptosis-related factors under mock-infected conditions. (A–D) Expression levels of PI3K-AKT pathway-related genes *CXCL10* **(A, B)** and *THBS4* **(C, D).**

(E–J) Expression levels of apoptosis-related genes *BAX* **(E, F)**, *CASP3* **(G, H)**, and *BCL-2* **(I, J).**

No significant differences were observed between the CTSC-overexpressing or CTSC-knockdown groups. Data are presented as mean ± SD from three independent experiments. n.s., not significant.
